# Genome comparison of two *Magnaporthe oryzae* field isolates reveals genome variations and potential virulence effectors

**DOI:** 10.1186/1471-2164-14-887

**Published:** 2013-12-16

**Authors:** Chenxi Chen, Bi Lian, Jinnan Hu, Huanchen Zhai, Xingxing Wang, RC Venu, Erming Liu, Zhilong Wang, Meilian Chen, Baohua Wang, Guo-Liang Wang, Zonghua Wang, Thomas K Mitchell

**Affiliations:** 1 Department of Plant Pathology, The Ohio State University, 2021 Coffey Road, Columbus, OH 43210, USA; 2 Key Laboratory of Bio-pesticide and Chemistry Biology, Ministry of Education, Fujian Agriculture and Forestry University, 15 Shangxiadian Road, Fuzhou, Fujian 350002, China; 3 Hunan Provincial Key Laboratory of Crop Germplasm Innovation and Utilization, College of Agronomy, Hunan Agricultural University, 1 Nongda Road, Changsha, Hunan 410128, China; 4 College of Bioengineering, Henan University of Technology, 100 Lianhua Ave., Zhengzhou, Henan 450001, China

**Keywords:** *Magnaporthe oryzae*, Next generation sequencing, Genome comparison, Candidate effectors identification, Isolate-specific genome content

## Abstract

**Background:**

Rice blast caused by the fungus *Magnaporthe oryzae* is an important disease in virtually every rice growing region of the world, which leads to significant annual decreases of grain quality and yield. To prevent disease, resistance genes in rice have been cloned and introduced into susceptible cultivars. However, introduced resistance can often be broken within few years of release, often due to mutation of cognate avirulence genes in fungal field populations.

**Results:**

To better understand the pattern of mutation of *M. oryzae* field isolates under natural selection forces, we used a next generation sequencing approach to analyze the genomes of two field isolates FJ81278 and HN19311, as well as the transcriptome of FJ81278. By comparing the *de novo* genome assemblies of the two isolates against the finished reference strain 70–15, we identified extensive polymorphisms including unique genes, SNPs (single nucleotide polymorphism) and indels, structural variations, copy number variations, and loci under strong positive selection. The 1.75 MB of isolate-specific genome content carrying 118 novel genes from FJ81278, and 0.83 MB from HN19311 were also identified. By analyzing secreted proteins carrying polymorphisms, in total 256 candidate virulence effectors were found and 6 were chosen for functional characterization.

**Conclusions:**

We provide results from genome comparison analysis showing extensive genome variation, and generated a list of *M. oryzae* candidate virulence effectors for functional characterization.

## Background

Rice has been served as a major food source for people in Asia and Africa for centuries. However a large portion of yield is lost through agriculture disease and pests annually [1]. Rice blast, caused by the fungal pathogen *Magnaporthe oryzae*, is one of the most severe rice diseases and has been found almost everywhere rice is grown [2,3]. Conidia of *M. oryzae* are transmitted by rain splash or plant-to-plant contact, and facilitate infection by penetrating into rice leaves using a specialized structure called an appressorium. Mycelia then extend through host tissue and causing cell death [4,5]. In the traditional gene-for-gene model, resistance (R) genes in the host specifically recognize corresponding avirulence (Avr) genes the pathogens. Recognition is followed by triggering a hypersensitive response (HR) [6]. This mechanism is the primary tool to control rice blast disease by introducing R genes into elite rice cultivars. However, such resistance can be broken within a few years of release [7,8], mostly due to the mutation or functional loss of the Avr genes.

Rice and *M. oryzae* have emerged as a model system for host-pathogen studies [9]. With the fast development of the next generation sequencing (NGS) technologies, genome re-sequencing and comparative studies have been reported in multiple fungal phytopathogens. Some common findings from these studies include a high level of variation among the genomes of different isolates and unique genome regions that carry virulence effectors [10-13]. One interesting finding from genome comparison projects was chromosome number variation, as was reported in *Fusarium oxysporum*[11], *Nectria haematococca*[14,15]*, Mycosphaerella graminicola*[16]*, Cochliobolus heterostrophus*[17]*, Leptosphaeria maculans*[18]*, and Alternaria alternata*[19,20]. One factor heading to chromosome number variation was the presence of small extra chromosomes. The small extra chromosomes observed were usually considered as supernumerary chromosomes or conditionally dispensable chromosomes (CDC), and were often associated with virulence [20]. In *M. oryzae*, the presence of a CDC was first reported in 1993 by Talbot [21], who analyzed chromosomes and found “minichromosomes” in 15 of the 19 field isolates collected from United States and identified CDCs ranging from 470 KB-2.2 MB in size. In another study, a 1.2 MB CDC was found in *M. oryzae* and size variation was observed in progeny [22]. In 2005, the *AvrPik* gene was found linked with a 1.6 MB CDC, which was then confirmed by contour-clamped homogenous field electrophoresis and Southern hybridization [23].

High throughput genome based studies have been performed on *M. oryzae* in the last decade. To identify novel Avr genes, a comparative genome study was conducted using isolate Ina168 from Japan. The major achievement of that study was that three Avr genes were identified and cloned [24]. Transcriptome libraries of another isolate Guy11 were used to identify genes associated with appressorium formation [25]. Recently, a comparative genome study, which included two field isolates, was published in 2012. The comparison reported isolate-specific genomic regions, genes under diversifying selection, and a large number of transposon-like elements with diversified sequences [26]. These successful studies using the NGS techniques demonstrate the feasibility and necessity of its application to *M. oryzae* field isolates to elucidate the molecular basis of virulence.

In this study, we performed whole genome sequencing on two *M. oryzae* field isolates FJ81278 and HN19311, as well as transcriptome sequencing on FJ81278. These are two field isolates collected from two different provinces in China (with a distance of approximately 900 km), and have been studied for years in two co-authors’ lab thus there are phenotypes and pathogenicity assay data available for these two isolates. The genome assemblies were compared to a sequence of reference strain 70–15. By conducting this analysis, we asked the following questions: 1) Do isolate-specific genes and genomic regions exist in these two field isolates? What are their functions and where are they originated? 2) How many variations can be found in comparison with 70-15? Do SNPs/indels serve as a major mutation sources? 3) How many genes that code for secreted proteins show polymorphisms and the potential to be virulence effectors? Here we report the results of analyses to answer these questions.

## Results and discussions

### Genome sequencing and assembly

Genomic DNA of isolates FJ81278 and HN19311 was prepared as paired-end libraries and sequenced by Illumina Genome Analyzer II at The Ohio State University. In total, 2.5GB of raw reads for FJ19311 and 475 MB reads for HN19311 were generated, which represented 34.0 ± 2.1 and 5.7 ± 0.2 sequencing depth, respectively. A hybrid *de-novo* assembly was performed, in which sequencing reads were first mapped to the reference genome of isolate 70–15 to generate a mapping file (in SAM format) to guide assembly, which were then combined with the unmapped reads and processed with the *de-novo* assembler Velvet with its new “Columbus module” [27]. As showed in Table 1, the two assemblies resulted in similar genome size around 37 MB, slightly smaller than the 70–15 reference genome. The FJ81278 assembly had 6,290 contigs with a N50 of 151.7 KB, while the HN19311 assembly had 6,249 contigs with a N50 of 147.4 KB. Long contigs (>5 KB) represented 92.8% of the FJ81278 assembly and 93.3% of the HN19311 assembly, suggesting the length of most contigs were suitable for open reading frame analysis.

**Table 1:** **The *de novo *** **assembly of two *M. oryzae *** **field isolates genome**

**Isolates**	**70-15**	**FJ81278**	**HN19311**
**Assembly contigs**	8	6,290	6,249
**Genome size (MB)**	39.4	37.3	37.1
**GC-content**	53.0%	51.3%	51.5%
**N50 length (KB)**		151.7	147.4
**Max contig length (KB)**		676.4	698.2
**Repeat elements (MB)**	3.60	1.02	1.05
**Size of contigs >5Kb (MB)**		34.78(92.8%)	34.62(93.31%)
**Unaligned contigs (MB)**		1.75	0.83

Reference 70–15 is included for comparison.

### Transcriptome assisted gene prediction

RNA-Seq reads were used in a similar way as traditional EST sequences to improve the quality and reliability of gene model predictions [28,29]. In this study, RNA-Seq reads from FJ81278 were utilized to accurately predict gene model structures, while in HN19311 an *ab initio* gene prediction was performed. The mRNAs for RNA-Seq were extracted from a mixture library covering different growth stages. In total, there were 63.1 million reads generated, which were first mapped to the FJ81278 assembly to generate hint files, and then gene structures were predicted by Augustus [30] assisted by the hint files. The PASA tool [31] was then used to adjust splice sites and expand the UTRs.

There were 10,453 genes predicted in the FJ81278 genome and 10,256 in HN19311. The gene count was approximately 2,000 fewer than that of sequenced strain 70–15 (Table 2). Although the average exon number per gene was almost identical among the three genomes, it should be noted that the average gene length was significantly shorter in HN19311, which was possibly caused by high fragmentation in the HN19311 assembly. Half of the FJ81278 genes received UTR annotations by comparing the transcriptome sequences and the coding regions.

**Table 2:** Statistics of gene prediction for the two isolates

**Isolate**	**70-15**	**FJ81278**	**HN19311**
**Mapped RNA-Seq reads**	-	6,280,625	-
**Predicted gene numbers**	12,827	10,453	10,256
**Average gene length (bp)**	2,003.9	2,085.0	1,745.4
**Average exons per gene**	2.68	2.76	2.73
**Gene density (per 10 KB)**	3.13	2.73	2.77
**3′-UTRs numbers**	-	5,824	-
**5′-UTRs numbers**	-	6,729	-

HN19311 gene prediction was performed *ab initio*, and FJ81278 gene prediction was assisted by RNA-Seq reads.

### Identification of presence/absence variation (PAV)

**Figure 1:**
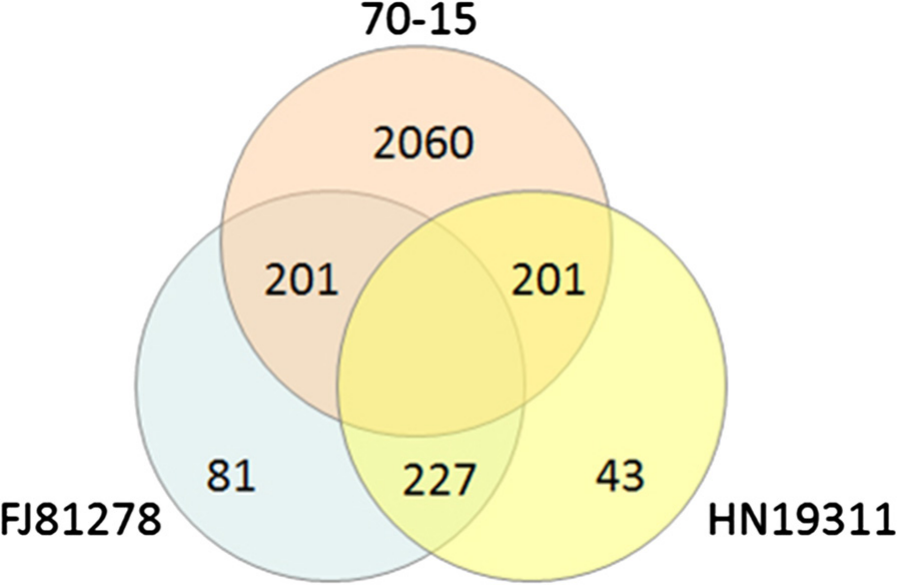
Venn diagram showing the presence/absence variation of the gene set from three strains.

To identify the unique genes in the two field isolates, the genomic content was compared to the 70–15 gene set. In order to avoid false positive gene predictions, the genes from each of the three gene sets (70–15, FJ81278, and HN19311) was aligned to genome sequences of the other two isolates using FASTA [32]. There were 195 genes from FJ81278 and 156 genes from HN19311 identified absent in 70–15 (Figure 1, see list in Additional file 1). Surprisingly, there were as many as 2,060 genes identified unique in 70–15, which may be the result of this isolate retaining genome content from the weeping grass parent it was originally developed from [33].

To gain a functional annotation of the unique genes in FJ81278 and HN19311, with the assumption that some of them will be involved in pathogenicity or environment adaptation, the gene sets were annotated by predicting secreted proteins. A large number of the secreted proteins were identified from the unique gene set: 62 (31.8%) from FJ81278 and 54 (34.6%) from HN19311, showing a enrichment of the secreted proteins in unique gene set.

The unique gene sequences were then input into BLAST tool to search against the NCBI “nr” database to annotate. Polyketide synthase (PKS) genes are known to be involved in fungal pathogenicity, and more than 20 of them were previously annotated in 70–15 [9]. We found 3 novel PKS genes from the unique gene sets. Six genes were identified as Protease with “A33” and “C48” as the top protease family; 17 transcription factors were identified from FJ81278 and 22 from HN19311; 7 genes from FJ81278 and 15 genes from HN19311 were identified as pathogenicity related genes based BLAST results to the PHI database [34] (E-value < 10^-5^). Finally, 13 “reverse transcriptases” were found, which can be part of transposons including two categories MGR583 and Pot2.

### Identification of SNPs and indels

Both of SNPs and indels are regarded as local genome region polymorphism indicators, and they can serve as high quality genome markers. To compare the two field isolates and the reference 70–15 at the nucleotide level, SNPs and indels were identified based on the 70–15 genome and annotations. As the result, 11,367 SNPs in FJ81278, with 24.4% in coding regions, and 5,666 SNPs in HN19311, with 24.2% in coding regions, were identified. FJ81278 and HN19311 had a similar SNP pattern of distribution regions (Additional file 2: Figure S1). There were 6,485 indels identified in FJ81278 and 1,372 identified in HN19311. The significantly fewer HN19311 SNPs and indels may be a result of lower sequencing depth. Interestingly, the distribution pattern of indels was different from the SNP pattern as more indels occurred in introns instead of exons (Additional file 2: Figure S2).

Although non-synonymous SNPs and indels in coding regions can have effects on associated proteins and sometimes alter the phenotype, the chance and level of the influence from SNPs or indels is hard to predict. On the other hand, those that caused a change at the start/stop codon and splice sites have large effects. After evaluating possible gene structure changes brought by each SNP in FJ81278 and HN19311, it was found that 104 genes contained SNPs located in start, stop codon or splice sites which causing large modification in protein sequences and thus altering their functions indel(Additional file 3: Table S1).

### Identification of structure variation

**Figure 2:**
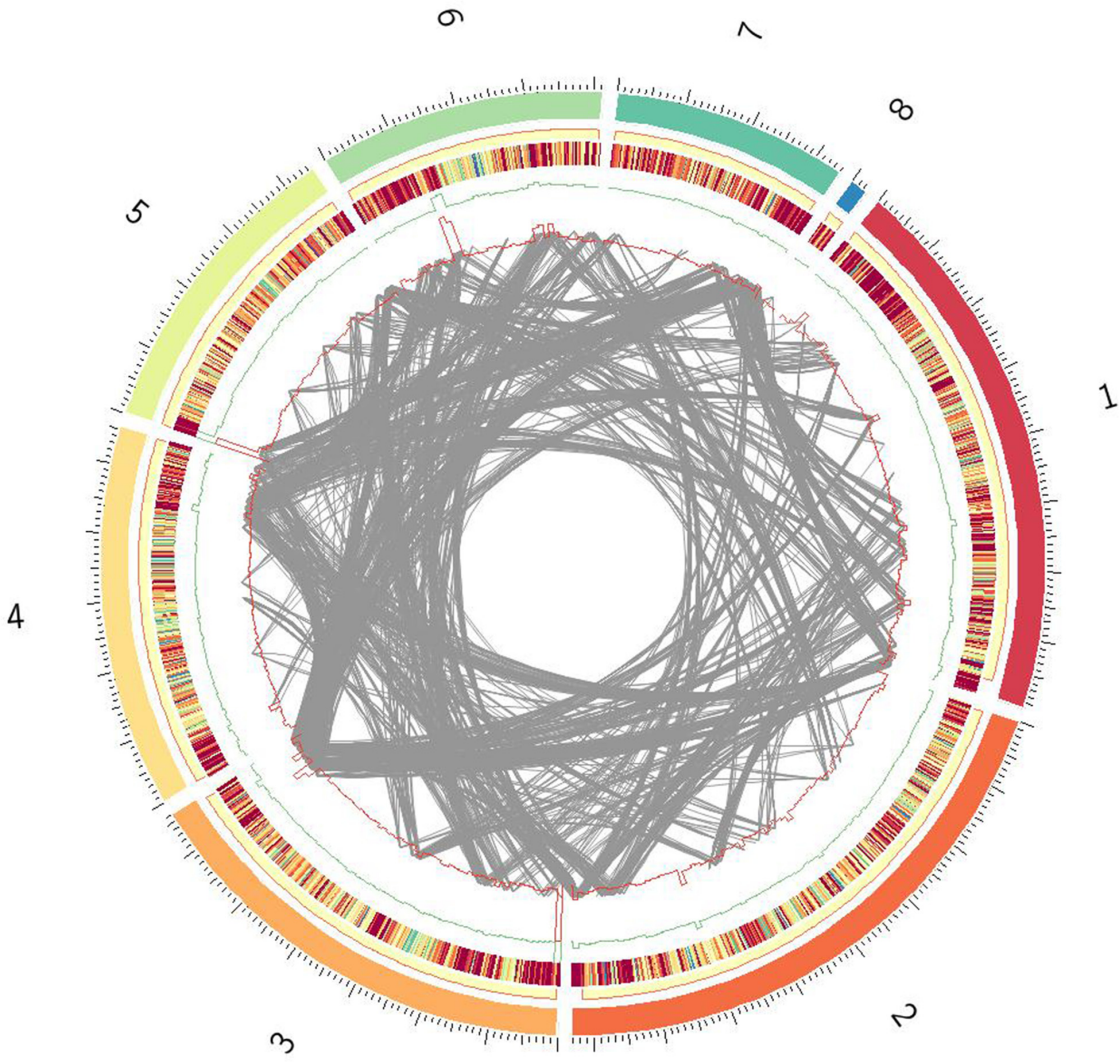
**Interchromosomal variation events found in FJ81278 genome.** Eight supercontigs (chromosomes) of 70–15 were circled and coded with different color. From outer to inner there are circle representing gene density with deeper red region showing higher gene density regions; green line and red line showing SNP density of FJ81278 and HN19311, respectly; and each grey line in the center part representing a possible translocation event scanned in a 500 – 1000 bp window.

Inter-chromosomal translocations, structural reconstructions, and chromosome length variations are more likely to be observed at the end of telomere regions, where most of the cloned Avr genes to date are located [35]. To evaluate the structure variation of FJ81278, possible inter-chromosomal translocation events were identified at the whole genome level by analyzing the mapping of paired-end reads (Figure 2). It can be observed that the majority of translocation events occurred in the telomere region, with a high density at the end of Supercontigs 8.3 and 8.4.

### Identification of copy number variation (CNV)

**Figure 3:**
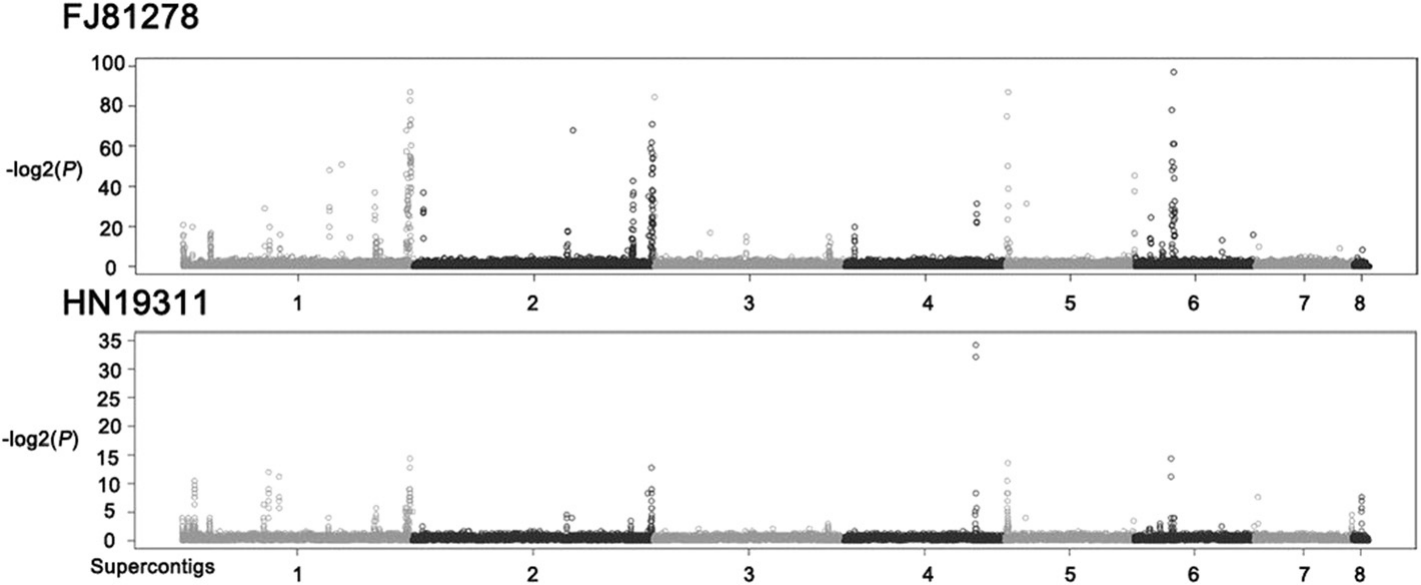
**Gene copy numbers along chromosomes for the two strains.** Peaks represent a possible CNV event.

Phytopathogens may increase the gene copy number of effectors in order to suppress host resistance. One reported example was for *P. sojae Avr1a*, *Avr3a*, and *Avr3c*, which were tandem repeats [36]. Another example was *Avr-Pita* gene in *M. oryzae*, which contained multiple copies on different chromosomes [35], leading to a hypothesis that multiple copies of effectors increases adaptation of pathogens. To estimate the CNV, mapped reads coverage of both isolates were scanned in a 1 KB window to identify CNV events based on a P-value calculated in a model of Poisson’s distribution. In FJ81278, major CNV events in the end of Supercontig 8.1, end of Supercontig 8.2, and 120-130 KB from one end in Supercontig 8.6, were found (Figure 3).

### Identification of genes under positive selection

Genes important in phytopathogen virulence may be associated with a rapid pattern of evolution for adaptation to new environments or hosts. To evaluate gene evolution rate and identify those under positive selection, the Ka/Ks ratios in the orthologous genes in FJ81278, HN19311, versus 70–15 were calculated. As showed in Table 3, 10 genes from FJ81278 and 7 genes from HN19311 had a Ka/Ks ratio > 1, indicating positive selection.

**Table 3:** Genes identified under positive selection and their functional annotation

**FJ81278**			**HN19311**		
**Gene ID**	**Ka/Ks**	**Descriptions**	**Gene ID**	**Ka/Ks**	**Descriptions**
**MGG_08542**	5.037	conserved hypothetical protein	**MGG_14967**	1.329	
**MGG_17751**	2.247	hypothetical protein	**MGG_14837**	1.301	
**MGG_15517**	1.765	hypothetical protein	**MGG_02059**	1.100	
**MGG_01447**	1.669	conserved hypothetical protein	**MGG_12484**	1.054	putative secreted protein
**MGG_12484**	1.467	putative secreted protein	**MGG_02289**	1.015	c6 finger domain
**MGG_02351**	1.418	tyrocidine synthase 1	**MGG_14894**	1.015	putative secreted protein
**MGG_15408**	1.283	hypothetical protein	**MGG_01447**	1.001	conserved hypothetical protein
**MGG_05595**	1.066	ABC transporter			
**MGG_03810**	1.054	polyketide synthase			
**MGG_12141**	1.030	COPII coat assembly protein SEC16			

Several genes identified in this group showed interesting functional annotation, for example, *MGG_08542*, was found to contain a NACHT domain and being annotated as “PCD (programed cell death) related”. The NACHT domain was also found in HET (Heterokaryon incompatibility protein) genes in most filamentous fungi [37], which in this study showed a high frequency of nucleotide diversity with 16 non-synonymous loci found in FJ81278 and HN19311. *MGG_15067* encoded the *HET* protein in *M. oryzae* and has been reported to prevent heterokaryon formation between genetically different individuals by inducing PCD [37]. The fact that the HET gene was driven by positive selection and had high sequence diversity in some fungal species indicated a regulatory function of the self/non-self-recognition system to facilitate independent individual heredity [38]. An ABC transmembrane transporter *MGG_05595* was also identified, which may function in infection as transporting toxins from the host (such as ROS) and thus contribute to resistance of *M. oryzae* to defenses. Genes containing ABC transmembrane transporter domains were found to be under positive selection in many organisms [39,40], with the *M. oryzae* ABC3 gene knockout mutant shown to be highly sensitive to fungicides and had a loss of pathogenicity [41].

### Identification of isolate-specific genome regions

As been mentioned in the introduction, several studies have found supernumerary chromosomes or isolate-specific genome regions in *M. oryzae.* In this study, comparison of genome content of these two isolates was performed by aligning the assembly contigs to the 70–15 reference sequences, from which 1.75 MB unaligned contigs from FJ81278 and 0.83 MB from HN19311 isolate were identified. The size of specific contigs were similar to that of supernumerary chromosomes previously reported (440 K-2.2 MB) [21]. Since no coding region was detected from the HN19311 isolate-specific region, the focus was placed on FJ81278 for the unique contig analysis.

The isolate-specific contigs in FJ81278 carried 118 coding genes, in which 27 were novel secreted proteins. Majority of these 118 genes showed no homology in 70–15. Forty-eight genes having orthology in 70–15 genome were identified, but no synteny block around those orthologous genes was found. The codon usage bias of specific contigs by calculating GC-content, GC3-content [42], and CAI (Codon Adaptation Index) [43] and comparing to the 70–15 reference genes was then analyzed. Significant difference in GC-content (*t*-test, P-value = 9.342e-12), GC3-content (P-value = 4.178e-10) (Additional file 2: Figure S3), and CAI (Additional file 2: Figure S4) from reference genes was found, suggesting a possible different evolutionary origin.

### Candidate effector identification

During the infection process, hosts resistance is suppressed by the pathogen effectors, which are usually small secreted proteins, and may contain conserved domains, such as the RxLR domain in oomycetes [44]. However, no conserved domains have been identified in cloned *M. oryzae* Avr genes, but high sequence variation was found instead [45-49]. In total, 1,243 proteins from all three isolates containing predicted signal peptides were identified, among which candidate effectors were predicted in the two field isolates based on the polymorphisms found in at least one of these five categories: (1) Presence/absence variation; (2) SNPs/indels; (3) Copy number variation; (4) Selection force; (5) TE insertion polymorphism.

There were 196 secreted proteins identified in the field isolate unique gene sets. From the SNP analysis, 58 secreted proteins from both isolates, and 68 FJ81278 unique secreted proteins were identified containing at least one non-synonymous SNP (Additional file 1). Importantly, this gene set included two known Avr genes: *AvrPik* and *AvrPita1*. The indel analysis showed a similar result as 64 proteins from FJ81278 and 43 proteins from HN19311 contained at least one indel locus, while 19 of them were shared by two isolates. Eighteen secreted proteins containing CNVs and 2 under positive selection were also found. Since TE insertion may serve as a major mechanism to break rice resistance, the TE insertion loci and their effected secreted proteins in FJ81278 were evaluated. HN19311 was not included in this analysis due to the lack of sufficient sequencing depth. As the result, 64 secreted proteins in FJ81278 containing TE insertion within 1500 bp upstream region were found (Additional file 1). Any gene landing in at least one of the five categories of polymorphisms discussed above was placed in the candidate effector set, which included 256 non-redundant genes.

### Functional analysis of candidate effector genes

Six secreted proteins from the FJ81278 unique gene set, which are also presented in the candidate effectors list, were randomly chosen to perform overexpression analyses (Table 4). Lengths of these 6 genes are < 1 KB and predicted to contain signal peptides. Among them, *g10399* was annotated as a “Cytochrome P450” and *g10338* was annotated as a “Methyl Transferase”, while the other 4 genes showed no homology to any protein in GenBank.

**Table 4:** The six FJ81278 unique secreted proteins chosen for functional characterizations

**Gene**	***g10399***	***g10338***	***g10395***	***g2480***	***g1914***	***g10396***
**Length (bp)**	490	594	810	585	383	291
**Subcellular Location**	cytoplasmic	Plasma Membrane	N/A	Extracellular	Extracellular	cytoplasmic
**Functional Annotation**	P450	Methyl transferase	Unknown	Unknown	Unknown	Unknown

**Figure 4:**
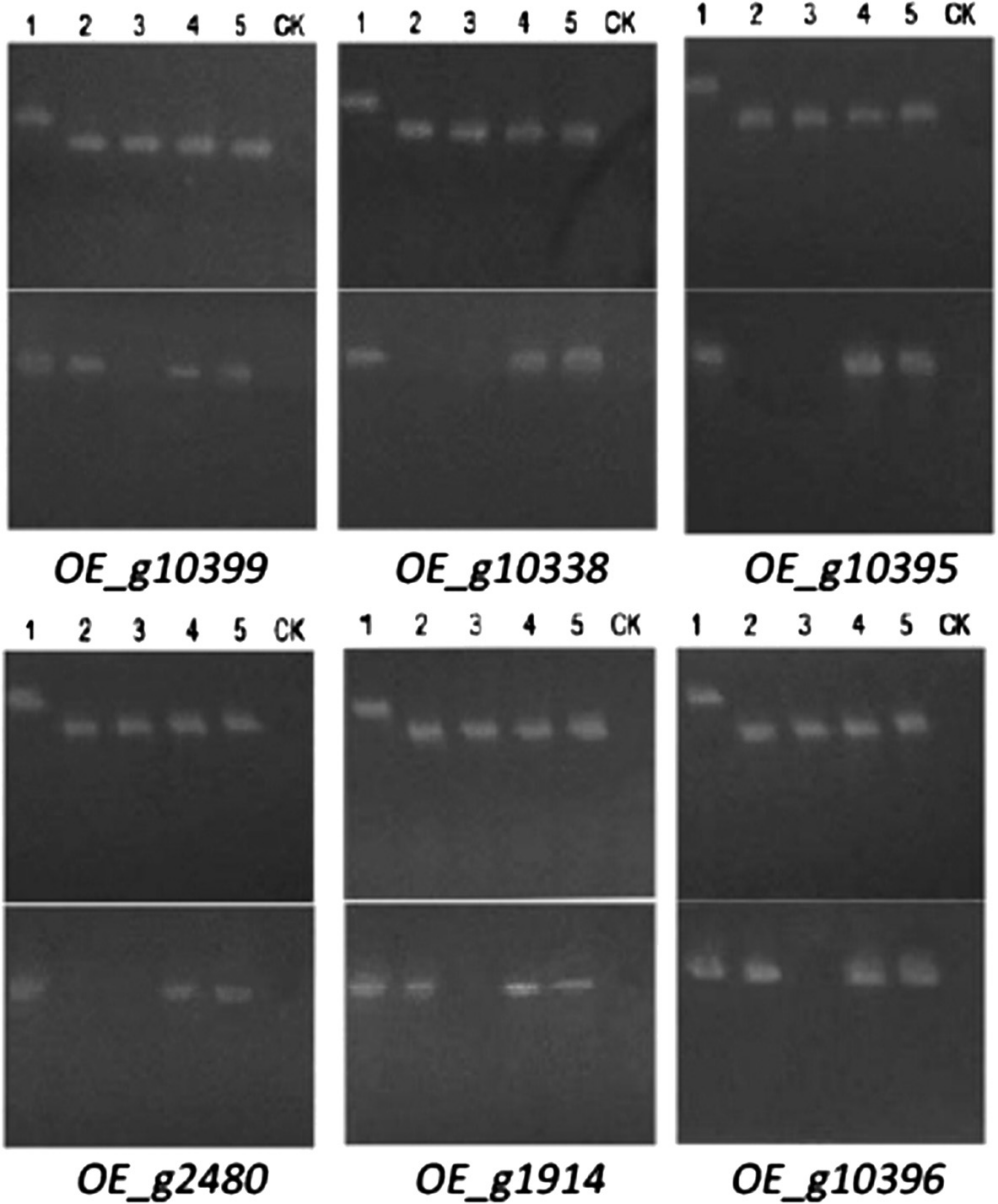
**Confirmation of GUY11 overexpression transformants by semi-quantitative PCR.** Lane 1: FJ81278 genomic DNA; Lane 2: FJ81278 cDNA; Lane 3: GUY11 cDNA; Lane 4 & 5: overexpression transformants cDNA; Lane CK: water*.* For each gene, the upper gel shows the “*actin*” control, and lower gel shows the semi-quantitative PCR result.

Multiple overexpression transformants were generated and PCR of the target genes were performed to confirm stable transformation. A total of 17 transformants of *g10399*, 5 of *g10338*, 9 of *g10395*, 5 of *g2480*, 4 of *g1914*, and 6 of *g10396* were obtained (Additional file 2: Figure S5). The expression level of these 6 genes with native promoters may vary depending on growth stage in wild type, but is supposed to maintain high expression levels in all conditions with the overexpression promoter provided (pDL1 Vector). Semi-quantitative PCR was conducted on the mRNA extracted from transformant mycelia, with *Actin* and water being used as a control. As showed in Figure 4, all the tested genes in transformants were stably expressed, while three of them (g10338, g10395, g2480) were barely expressed driven by their native promoters in FJ81278.

**Figure 5:**
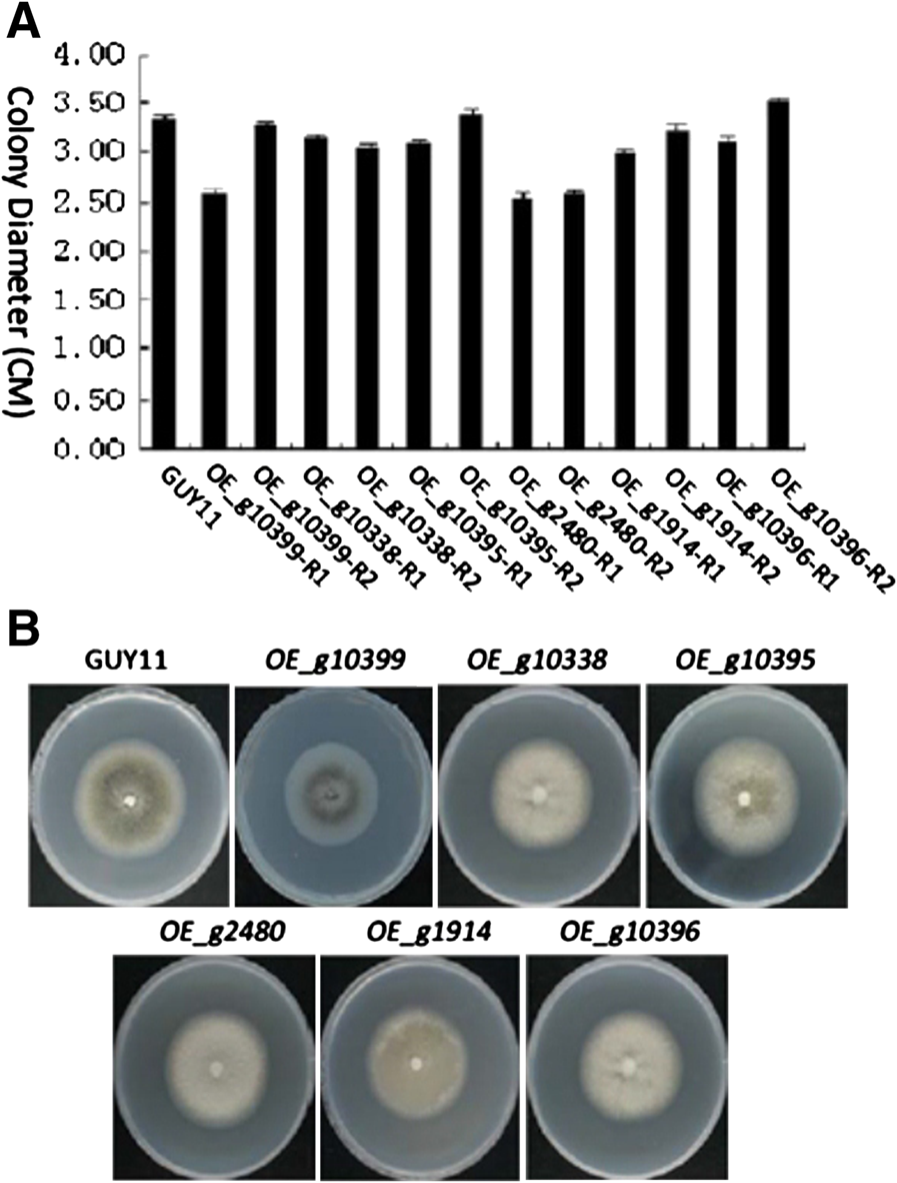
**Growth rate test of overexpression transformants and GUY11 wild type. (A)**: Growth rate of transformants, two isolates for each gene. **(B)**: Pictures of typical transformants colonies.

Two transformants were randomly picked for each of the 6 genes and characterized for their growth, sporulation, germination, appressorium formation, penetration on onion epidemic cells, and virulence on a panel rice cultivars. *OE_g2480* overexpression mutants showed a slower rate, with only 75% in colony diameter compared to wild type. Although some transformants showed slightly slower rate compared to wild type, there was no significant difference observed (Figure 5A). It was observed that *OE_g10399* mutants had a significant color change between its center versus edge, which did not exist in any of other colonies (Figure 5B).

Sporulation (Additional file 2: Figure S6A) of transformants showed a high degree of variations, as *OE_g10395*, *OE_g2480*, and *OE_g10396* all showed higher rate of sporulation, and *OE_g10395* was almost doubled that of wild type. It should also be noted that *OE_g1914* showed decreased sporulation. Germination rate (Additional file 2: Figure S6B) was calculated 4 hours after dropping conidia suspension on a parafilm, and the appressorium formation rate (Additional file 2: Figure S6C) was calculated after 12 hours. Although *OE_g10399* and *OE_g1914* showed a decreased percentage (around 10%) in both tests, no significant difference between wild type and transformants was observed. Infection rate on onion epidemic cells (Additional file 2: Figure S6D) was calculated 24 hours after inoculation. Again, a decreased rate was observed in *OE_g10399* (75.3%) and *OE_g1914* (70.5%) compared to wild type (%), with the other four transformants also showing slightly decreased infection rates.

In summary, *OE_g10399* and *OE_g1914* both showed lower rates in colony growth, conidia germination, appressorium formation, and onion epidemic cell infection, but not sporulation, suggesting these two genes might be involved in vegetative growth.

Pathogenicity assays were performed using 2 transformants for each overexpression line and 6 rice cultivars containing different resistant genes including CO39, C101lAC *Pi-1*(t), C101A51 *Pi-2*(t), C104PKT *Pi-3*(t), C101PKT *Pi-4a*, C101TTP-4 L-23 *Pi-4b*. As showed in Additional file 2: Figure S7, all transformants and wild type GUY11 had no difference in all of the inoculations, eliminating the possibility that any of these 6 genes serves as *Avr-Pi1*, *Avr-Pi2*, *Avr-Pi3*, or *Avr-Pi-4*. Transformants were spray-inoculated onto eight additional rice cultivars including IRBLb-B(*Pi-b*), IRBLkm-Ts(*Pi-km*), IRBLkh-K3(*Pi-kh*), IRBLz-Fu(*Pi-z*), IRBLt-K59(*Pi-t*), IRBLz5-CA(*Pi-z5*), irblzt-T(*Piz-t*), and IRbL7-M(*Pi-7*(t)). All transformants showed identical virulence to that of wild type GUY11 (data not shown).

## Conclusion

In this study, by applying next generation sequencing we generated the *de-novo* assemblies of two *M. oryzae* field isolates FJ81278 and HN19311. The genome variation was estimated at both the nucleotide and chromosome region levels by comparing them against the genome of the reference isolate 70–15. Isolate-specific genes and isolate-specific genome regions were identified, which may originate from different species other than *M. oryzae*. SNPs/indels, CNVs, and structural variations were also analyzed, especially those enriched at coding regions and telomeric regions. The Ka/Ks ratio was scanned along the genome, leading to the identification of some key genes under positive selection. Finally, we identified 256 candidate effectors and chose 6 for functional characterization. Several phenotype and virulence differences were identified as compared to wild type. However, the remaining genes are the focus of continued investigation.

## Methods

### *M. oryzae* field isolates used

Field strain FJ81278 was collected at Fujian Province, China in the year 1981, and provided by Dr. Zonghua Wang in Fujian Agriculture and Forestry University, China. HN19311 was collected at Hunan Province, China in the year 2004 by Dr. Erming Liu in Hunan Agriculture University, China.

### Genome and transcriptome sequencing

Both isolates were inoculated in CM liquid media, and DNA was extracted following a protocol described [50]. The mRNA for transcriptome sequencing was extracted from FJ81278 isolate in developing stages including mycelium, conidia, and appressorium formation, which was then reverse transcribed into cDNA. Sequencing libraries were prepared using the Illumina Paired-End DNA sample Prep Kit and sequenced by Illumina Genome Analyzer II. The two genome libraries were barcoded with separate tags, then pooled to be sequenced in a single lane.

### Genome assembly

Short reads generated were mapped to the reference genome 70–15 (version 8, *Magnaporthe* Comparative Sequencing Project, Broad Institute of Harvard and MIT) supercontigs using SOAPaligner2 [51] with two mismatches allowed and insert length set as 100-300 bp. The alignment result was converted into SAM format and input into Velvet with the unmapped short reads together. Velvet Columbus module [27] was used to perform a reference guided *de-novo* assembly in which the SAM file was used to assign reads into their positions. The k-mer used by Velvet was optimized by test running and eventually determined at 47 bp for both isolates.

### Gene structure prediction

Gene structures of both isolates were first generated by using *ab initio* predictor Augustus [30] with pre-trained parameter for *M. gris*ea. Then FJ81278 gene structures were refined by using transcriptome sequencing data. All the RNA-Seq reads were first mapped to FJ81278 genome assembly using Tophat [52] which allowed gaps in alignment to span introns. Given the high sequencing depth in RNA-Seq, no mismatch was allowed in mapping to increase accuracy. Then the mapping file was processed by a transcriptome assembler “Inchworms”, which is now a module of transcriptome assembler “Trinity” [53], to *de-novo* assemble into transcript contigs. The output sequences were then filtered by Seqclean (http://compbio.dfci.harvard.edu/tgi/software/) to remove low complexity sequences and vector contaminations. As the last step, cleaned transcript sequences were input into PASA [31] to update and refine the predictions from Augustus, and UTRs were added at this step.

### Isolate-specific gene identification

This gene comparison was performed to identify unique genes that did not exist in genome content of other isolates. To reduce false positives due to variation in gene prediction process, gene sequences were aligned against genome sequences of other isolates rather than directly comparing gene sequences. The tool FASTA (version 35) [32] was used to BLAST gene set sequences. Then genes with low opt score (<200) were considered as “isolate-specific” genes.

### SNPs/indels identification

Genomic sequencing reads from field isolates were first mapped to the genome of reference strain 70–15 with TE region masked, using SOAPaligner2 [51] and then the sorted alignment files were input into SOAPSnp [54] for SNP identification with parameter set as “r 0.0001 –t –u –L 76”. In the filtering process, any SNP with less than 90% supported reads, coverage less than 2 or higher than 100 were filtered out. To identify indels, a pipeline including SAMtools [55] and BCFtools (http://samtools.sourceforge.net/mpileup.shtml) was used to process alignment files. All identified indels were supported by at least 8 reads. Then annotation of SNPs/indels was performed by snpEff [56] based on 70–15 gene sets.

### Structure variation identification

Structure variation in this study was detected by “abnormal” pared-end reads, which showed different mapping distance between the paired reads other than designed insert length of sequencing library. We consider the insert length of all paired-end reads followed Poisson distribution. Paired-end reads with insert length longer than the high-end cut-off indicate a deletion event; reads with insert length shorter than the low-end cut-off indicate an insertion event; reads mapped to the same direction of reference indicate inversion event; reads mapped to different chromosomes indicate an inter-chromosomal structural variation. The circle map visualizing the translocation events was drawn using Circos [57].

### CNV identification

Sequencing coverage along genomes distribute was considered as Poisson distribution. The sequencing coverage was scanned in a 1000 bp window along genome. A CNV was identified if one or multiple contiguous windows showed sequencing coverage significantly higher than genome median.

### Selection force calculation

Genes were aligned in pairs between each two gene set of FJ81278, HN19311, and 70–15. Codeml tool in PAML suite [58] was used to calculate Ka/Ks ratio, with the assumption that Ka/Ks ratio > 1 suggested the gene was under positive selection. We used different models – M1a, M2a, M7, M8 - in Codeml calculating to avoid bias.

### TE insertion detection

Given the short read length of reads generated, the method described by Kofler [59] was applied to detect distribution of TE insertion in FJ81278. Reference genome sequences were first processed by RepeatMasker [60] to mask TE regions, and the masked sequences were extracted to form a separate TE sequence sets. Then paired-end reads were mapped to the reference genome and TE sequence sets, respectively. If one of the paired-end read mapped to the reference genome and the other read mapped to TE sequences then it may indicate a TE insert loci. Every identified locus was supported by at least 3 paired-end reads.

### Overexpression transformation

Primers were designed and PCR was used to amplify the 6 chosen genes (Additional file 3: Table S2). Their ORF sequences were ligated to vector pTE11, and the positive ligations were confirmed by *SwaI* digestion as well as Sanger sequencing. Ligation products were transferred into yeast, and overexpression vector was generated by homologous recombination. The yeast plasmids were extracted and transferred into *E. coli* competent cells. The correctly inserted plasmids were confirmed by repeated PCR and sequencing, and then transferred in to GUY11 protoplasts. Colonies growing on selection media (300-400 mg/ml *HygB*) after 5–10 days in dark condition were picked.

### Phenotype characterization

To calculate the growth rate, fresh mycelium cubes were placed at the center of yeast extract media plate, and kept upside-down at 28°C condition for 10 days. Colony diameters of each transformants were counted with three repeats. Sporulation number was calculated by counting the spore number in 2 ml ddH_2_O from 7 days oatmeal plates. Then the spore concentration was adjusted to 1×10^4^-3×10^4^/ml and dropped on the hydrophobic surface of a parafilm. Germinated spores were counted after 4 hours and germination rate was calculated. The appressorium formation rate was calculated after 12 hours. Infection of onion epidemic cells was checked 24 hours after inoculation.

### Pathogenicity assay

Transformants were inoculated on oatmeal media for 7 days in dark condition and 28°C. Mycelia were then scratched off with sterilized blades and the media plates stayed under continues light condition for 2–3 additional days for sporulation. Spores were collected the washed by 0.02% Tween20 solution and filtered through 2-layer miracloth. The concentration of spore suspension was adjusted to 1.0-2.5×10^5^/ml for spray inoculation on rice seedlings at 3–4 leave stage. Each pot of seedlings was inoculated with 20 ml spores suspension and kept in wet for 24 hours, and then stayed in green house for 7 days. Lesions were checked and categorized into six levels according to the protocol and standard as described by Valent [61].

## Availability of supporting data (not uploaded yet)

The whole genome shotgun project has been deposited at DDBJ/EMBL/GenBank under the accession ATNU00000000 for FJ81278 genome assembly, and ATNT00000000 for HN19311 genome assembly and FJ81278 RNA-Seq short reads has been deposited into GenBank SRA database under sample SRS453988 and experiment SRX316682.

## Abbreviations

Avr: Avirulence; HR: Hypersensitive; NGS: Next generation sequencing; CDC: Conditionally dispensable chromosome; PAV: Presence/absence variation; PKS: Polyketide synthase; CNV: Copy number variation; PCD: Programmed cell death; HET: Heterokaryon incompatibility protein; CAI: Codon adaptation index; SNP: Single nucleotide polymorphism.

## Competing interests

The authors declare that they have no competing interests.

## Authors’ contributions

CC, study designing and writing, RNA-Seq assisted gene prediction, effectors identification; BL, bioinformatics analysis, manuscript preparation; JH, study designing, manuscript preparation; HZ, transcriptome preparation and analysis; XW, overexpression transformation, functional characterization; RV, sequencing library; EL, ZW, HN19311 isolate; MC, phenotypic analysis of transformants; BW, FJ81278 isolate, transformation; GW, study designing, manuscript preparation; ZW, study designing, manuscript preparation; TM, conceived and designed study, analysis, and writing. All authors read and approved the final manuscript.

## Supplementary Material

Additional file 1 Supplementary file gene list table contains: the FJ81278 unique gene list; secreted gene list with non-synonymous SNPs in FJ81278; list of FJ81278 genes with TE insertions in promoter regions; secreted gene list with non-synonymous SNPs in HN19311. Click here for file

Additional file 2: Figure S1, Figure S2, Figure S3, Figure S4, Figure S5, Figure S6, and Figure S7 **Figure S1.** Distribution of SNPs in different genome feature annotations. **Figure S2.** Distribution of indels in different genome feature annotations. **Figure S3.** Difference of 70–15 gene and FJ81278 unique genes in GC3-content. **Figure S4.** Difference of 70–15 gene and FJ81278 unique genes in Codon Adaptation Index. **Figure S5.** PCR identification of overexpression transformants. **Figure S6.** Phenotype characterization of the overexpression transformants. **Figure S7.** Pathogenicity assay of overexpression transformants and wild types on different rice cultivars. Click here for file

Additional file 3: Table S1 and Table S2 ** Table S1.** Number of proteins largely effected by SNPs/indels. **Table S2.** Primers used for PCR amplification during overexpression transformation. Click here for file
