# Supplementary material for: Genome comparison of two Magnaporthe oryzae field isolates reveals genome variations and potential virulence effectors
Source: BMC Genomics. 2013 Dec 16;14:887. doi: 10.1186/1471-2164-14-887 (PMC3878650; doi:10.1186/1471-2164-14-887)

Supplementary figures

Figure S1. Distribution of SNPs in different genome feature annotations.

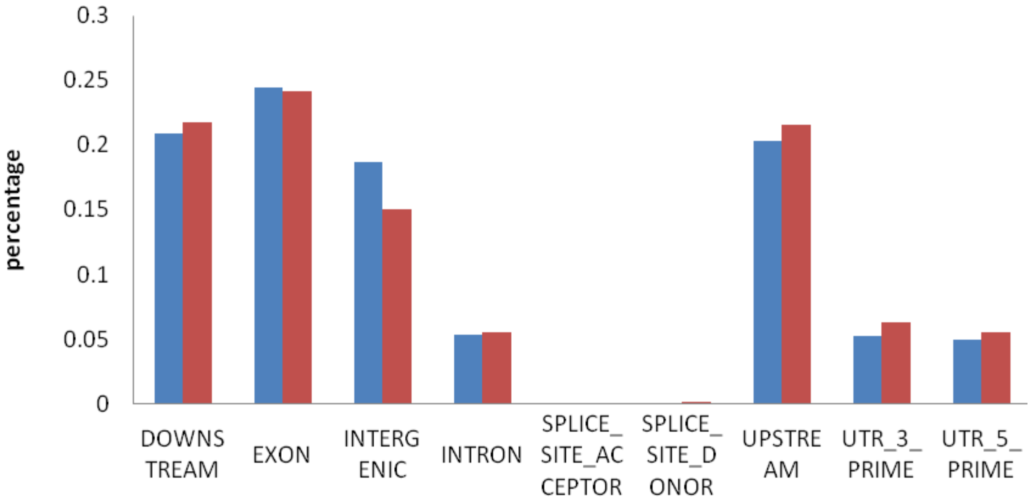

	DOWNSTREAM	EXON	INTERGENIC	INTRON	SPLICE_SITE_ACCEPTOR	SPLICE_SITE_DONOR	UPSTREAM	UTR_3_PRIME	UTR_5_PRIME
FJ81278	0.20926	0.24429	0.18691	0.05366	0.00035	0.00084	0.20275	0.05282	0.04911
hn19311	0.21749	0.24174	0.15061	0.05495	0.00027	0.0011	0.21598	0.06249	0.05537

Figure S2. Distribution of Indels in different genome feature annotations.

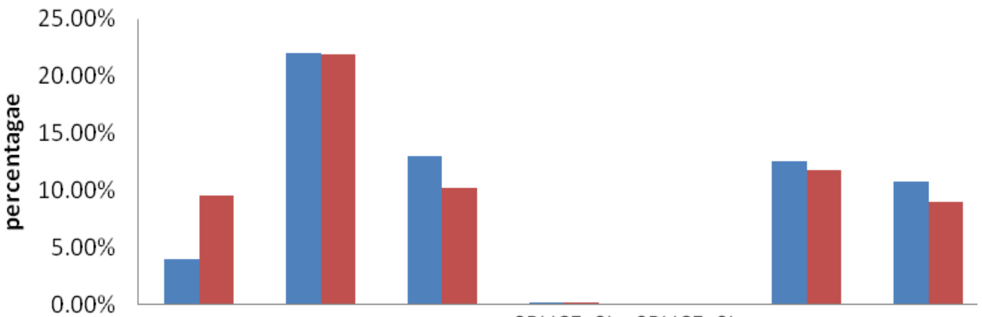

	EXON	INTERGENIC	INTRON	SPLICE_SITE_ACCEPTOR	SPLICE_SITE_DONOR	UTR_3_PRIME	UTR_5_PRIME
FJ81278	3.99%	21.96%	12.98%	0.21%	0.04%	12.49%	10.76%
HN19311	9.52%	21.85%	10.19%	0.18%	0.06%	11.79%	8.96%

Figure S3. Difference of 70-15 genes and FJ81278 unique genes in GC3-content.

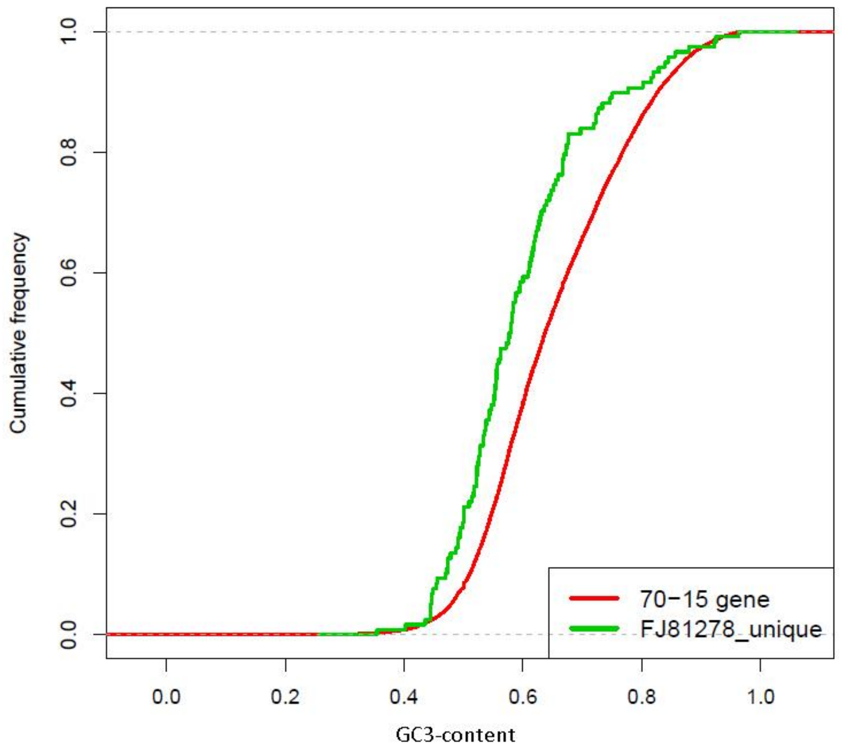

Figure S4. Difference of 70-15 genes and FJ81278 unique genes in Codon Adaptation Index.

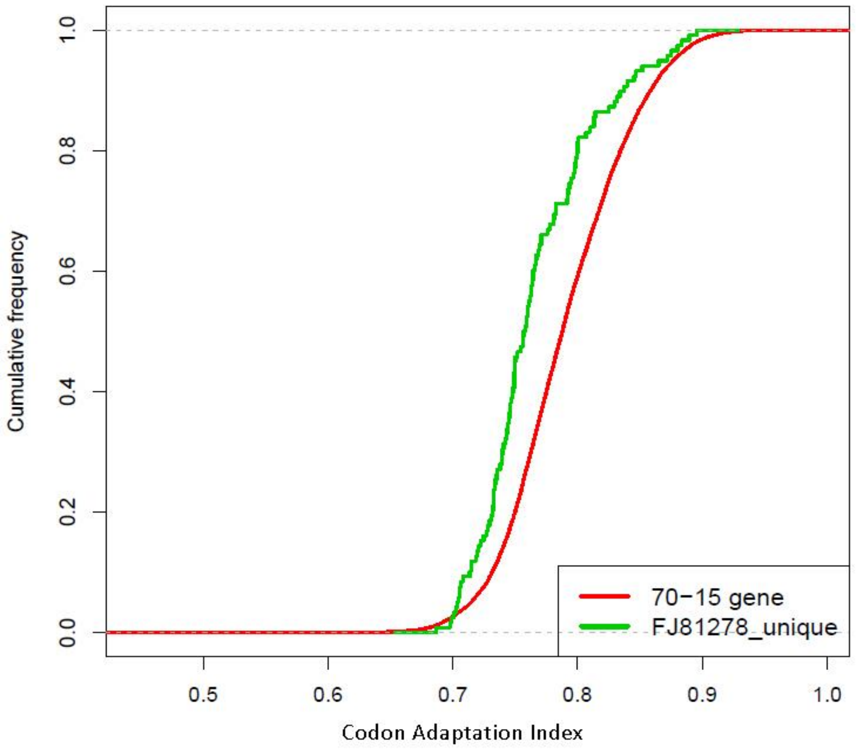

Figure S5. PCR identification of overexpression transformants. "M": Marker; "+": FJ81278 genomic DNA; "G": GUY11 genomic DNA; Number: *Hyg* resistant transformants; "CK": H_2O.

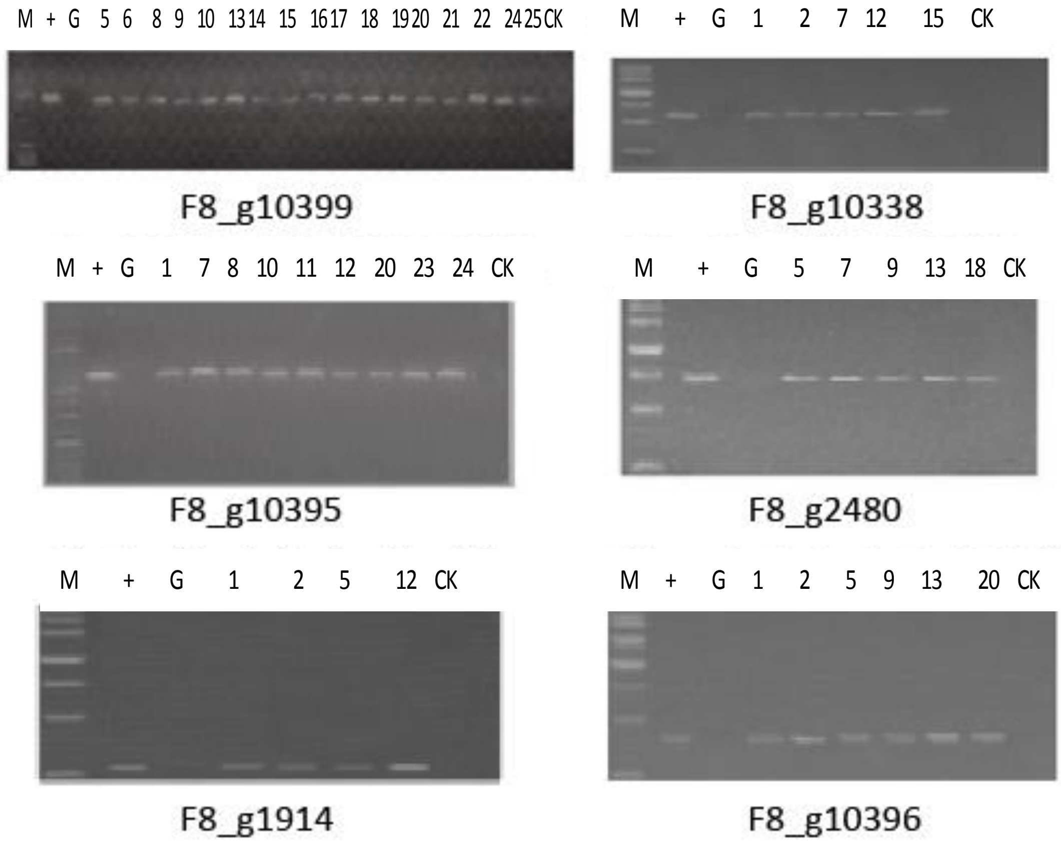

Figure S6. Phenotype characterization of the overexpression transformants. (A) Sporulation rate; (B) Spore germination rate on hydrophobic surface; (C) Appressorium formation rate on hydrophobic surface; (D) Infection assay on onion epidemic cells.

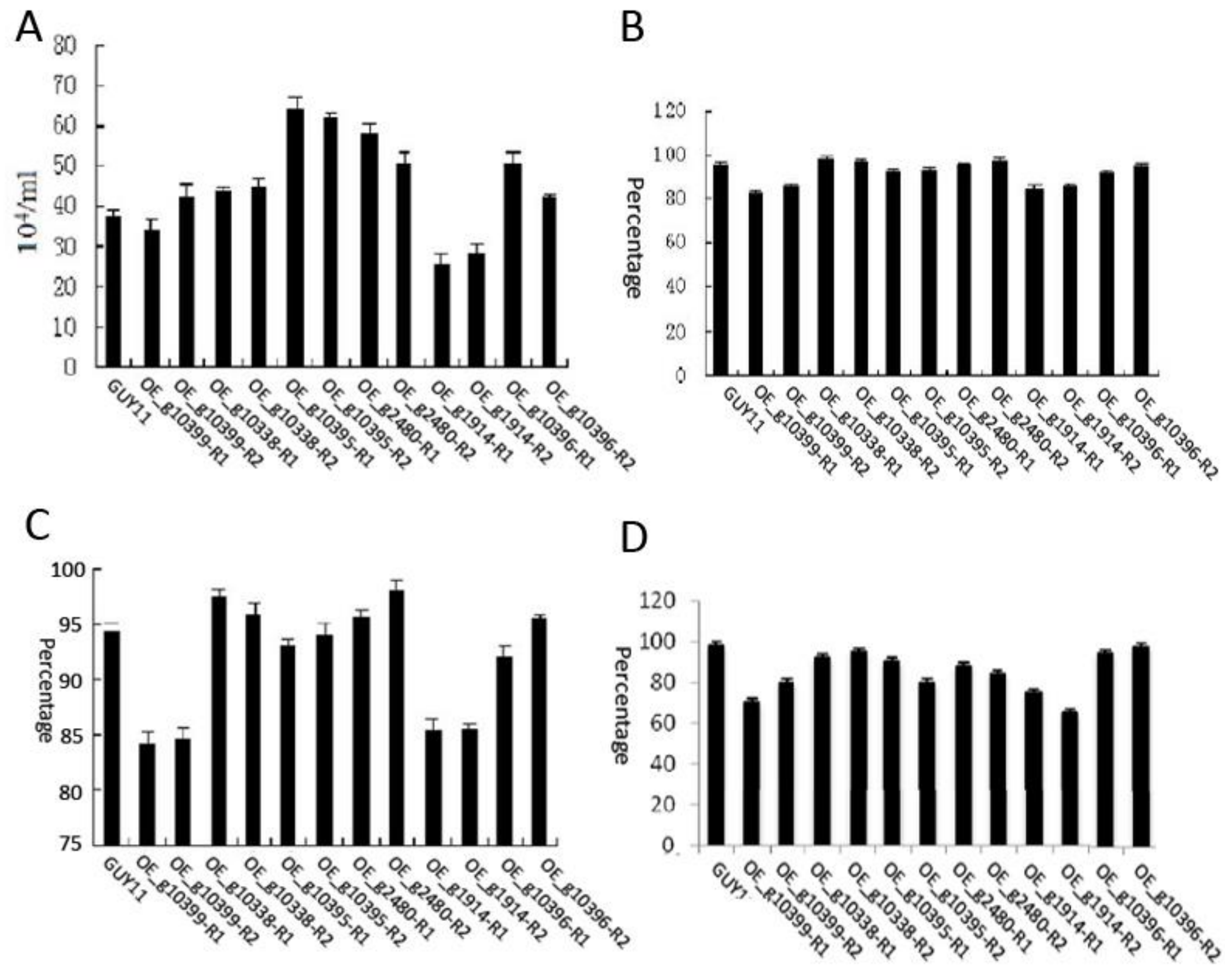

Figure S7. Pathogenicity assay of overexpression transformants and wild types on different rice cultivars. (A) Summary of the pathogenicity assay; (B) Selected Infections of two wild types and two overexpression transformants.

A

Strain	CO39	*Pi-1*	*Pi-2*	*Pi-3*	*Pi-4a*	*Pi-4b*
GUY11	Vir	Vir	Vir	Vir	Vir	Avr
FJ81278	Vir	Avr	Avr	Vir	Avr	Avr
OE_g10399-R1	Vir	Vir	Vir	Vir	Vir	Avr
OE_g10399-R2	Vir	Vir	Vir	Vir	Vir	Avr
OE_g10388-R1	Vir	Vir	Vir	Vir	Vir	Avr
OE_g10338-R2	Vir	Vir	Vir	Vir	Vir	Avr
OE_g10395-R1	Vir	Vir	Vir	Vir	Vir	Avr
OE_g10395-R2	Vir	Vir	Vir	Vir	Vir	Avr
OE_g12480-R1	Vir	Vir	Vir	Vir	Vir	Avr
OE_g12480-R2	Vir	Vir	Vir	Vir	Vir	Avr
OE_g1914-R1	Vir	Vir	Vir	Vir	Vir	Avr
OE_g1914-R2	Vir	Vir	Vir	Vir	Vir	Avr
OE_g10396-R1	Vir	Vir	Vir	Vir	Vir	Avr
OE_g10396-R2	Vir	Vir	Vir	Vir	Vir	Avr

B

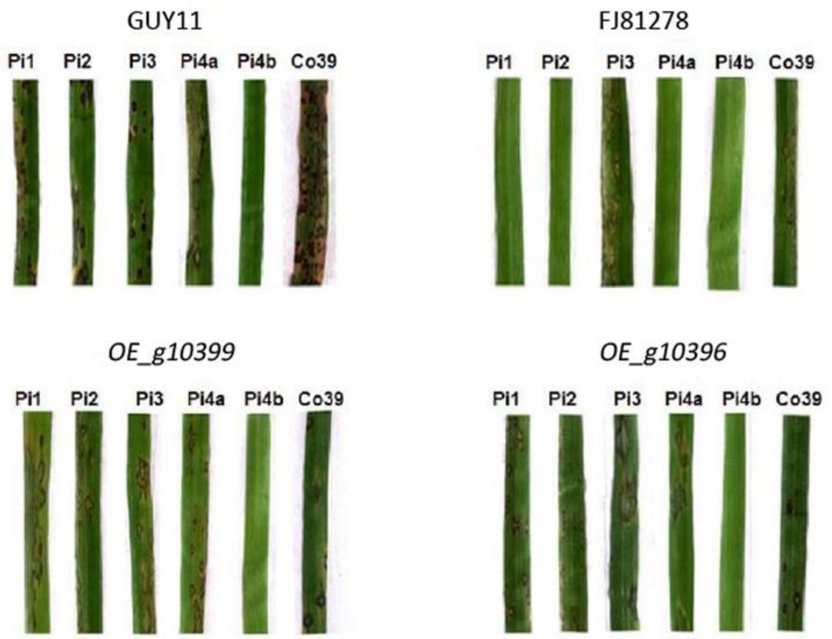

Supplement: Additional file 2: Figure S1, Figure S2, Figure S3, Figure S4, Figure S5, Figure S6, and Figure S7 — Figure S1. Distribution of SNPs in different genome feature annotations. Figure S2. Distribution of indels in different genome feature annotations. Figure S3. Difference of 70–15 gene and FJ81278 unique genes in GC3-content. Figure S4. Difference of 70–15 gene and FJ81278 unique genes in Codon Adaptation Index. Figure S5. PCR identification of overexpression transformants. Figure S6. Phenotype characterization of the overexpression transformants. Figure S7. Pathogenicity assay of overexpression transformants and wild types on different rice cultivars. [file 1471-2164-14-887-S2.pdf]
