# Supplementary material for: Genome comparison of two Magnaporthe oryzae field isolates reveals genome variations and potential virulence effectors
Source: BMC Genomics. 2013 Dec 16;14:887. doi: 10.1186/1471-2164-14-887 (PMC3878650; doi:10.1186/1471-2164-14-887)
Supplement: Additional file 3: Table S1 and Table S2 — Table S1. Number of proteins largely effected by SNPs/indels. Table S2. Primers used for PCR amplification during overexpression transformation. [file 1471-2164-14-887-S3.pdf]

Supplementary tables

Table S1. Number of proteins largely affected by SNPs/Indels.

Type	Isolate	Splice Site Accepter	Splice Site Donor	Stop Codon Gained	Stop Codon Lost
**SNP**	FJ81278	5	12	24	15
	HN19311	2	8	11	4
**INDEL**	FJ81278	13	3	0	2
	HN19311	3	1	1	0

Table S2. Primers used for PCR amplification during overexpression transformation.

Primer ID	Sequences
g10399-F	CCGCTCGAGAATGTGGGATTACGAGATT
g10399-R	CGGGATCCCTAGTCGATAACTCTTCGGAC
g10338-F	CCGCTCGAGATCAGGCTATGGCGGTCCAAATGTA
g10338-R	CGGGATCCATGGCCCACAATATACGAGAAAA
g10395-F	CCGCTCGAGAATGCAGACTCAAAATCTCTTGG
g10395-R	CGGGATCCTCGGCTCAAGAAATATGAAAAC
g2480-F	AGGAACCCAATCTTCAAAATGCACACTTTCAAACTCTT
g2480-R	ATGGTGATGAGAACCACGGGCACCTGGATAGCTGGATC
g1914-F	AGGAACCCAATCTTCAAAATGCCACCCCCGACCGCTCC
g1914-R	ATGGTGATGAGAACCACGAGGCTTGCCGACGACGTCGG
g10396-F	AGGAACCCAATCTTCAAAATGCAGACTCAAAATCTCTT
g10396-R	ATGGTGATGAGAACCACGAATCGAGTCAACGTGTCCAT
